# Acute clouding of trifocal lens during implantation: a case report

**DOI:** 10.1186/s12886-017-0636-7

**Published:** 2017-12-08

**Authors:** Qian Liu, Suhua Zhang, Xiaogang Wang, Weifang Cao, Yading Jia

**Affiliations:** Shanxi Eye Hospital, No. 100 Fudong Street, Taiyuan, Shanxi 030002 People’s Republic of China

**Keywords:** AT LISA tri 839mp, Opacification, Cloudy

## Abstract

**Background:**

Intraoperative IOLs clouding of several kinds of hydrophilic acrylic intraocular lenses (IOLs) have been reported due to temperature changes. This phenomenon reported previously occurred in cold countries and during the winter months. However, no clinical case was reported about trifocal IOL opacification during operation. We report a case in which acute opacification of the optical region occurred simultaneously when AT LISA tri 839mp(Carl Zeiss) was implanted into the eye.

**Case presentation:**

A 79-year-old woman with a cortex and nucleus cataract was scheduled to undergo right eye phacoemulsification assisted by femtosecond technique. The trifocal lens (AT LISA tri 839mp), which is made of hydrophilic acrylic (25%) with hydrophobic surface properties, was chosen for implantation. As the IOL was implanted into the eye, it became cloudy immediately. Then it was replaced by another AT LISA tri 839mp, which was transferred from lens company outside, the same phenomenon was observed. These two lenses underwent the same temperature fluctuation from cold outside to operating room. Finally, a ZCB00 (Allergan) was implanted.

**Conclusions:**

The acute intraoperative clouding of trifocal lens(AT LISA tri 839mp) results from fluctuation of temperature should be noticed.

## Background

The major hydrophilic acrylic intraocular lenses(IOLs) that have been reported on regarding postoperative opacification include Hydroview (Bausch& Lomb), SC60B-OUV (Medical Developmental Research, Inc.), ACRL-60 (Ophthalmed, LLC), Memory Lens (Ciba Vision), AquaSense (Ophthalmic Innovations International, Inc.), and Akreos Adapt AO (Bausch & Lomb) [1–6]. Delayed opacification of IOLs is known as pseudocataract [7, 8]. Intraoperative acute clouding of acrylic hydrophilic IOLs has been reported [9]. There have been no reports of intraoperative acute clouding with the AT LISA tri 839mp.

## Case presentation

To meet patient expectations of high-quality postoperative vision, including distant, medium, and near vision, a 79-year-old woman with a cortex and nucleus cataract was scheduled to undergo right eye phacoemulsification assisted by femtosecond laser technique with a + 18.5diopter trifocal IOL(AT LISA tri 839mp, Carl Zeiss)implantation. Her preoperative best-corrected visual acuity was logMAR 0.3.

**Fig. 1:**
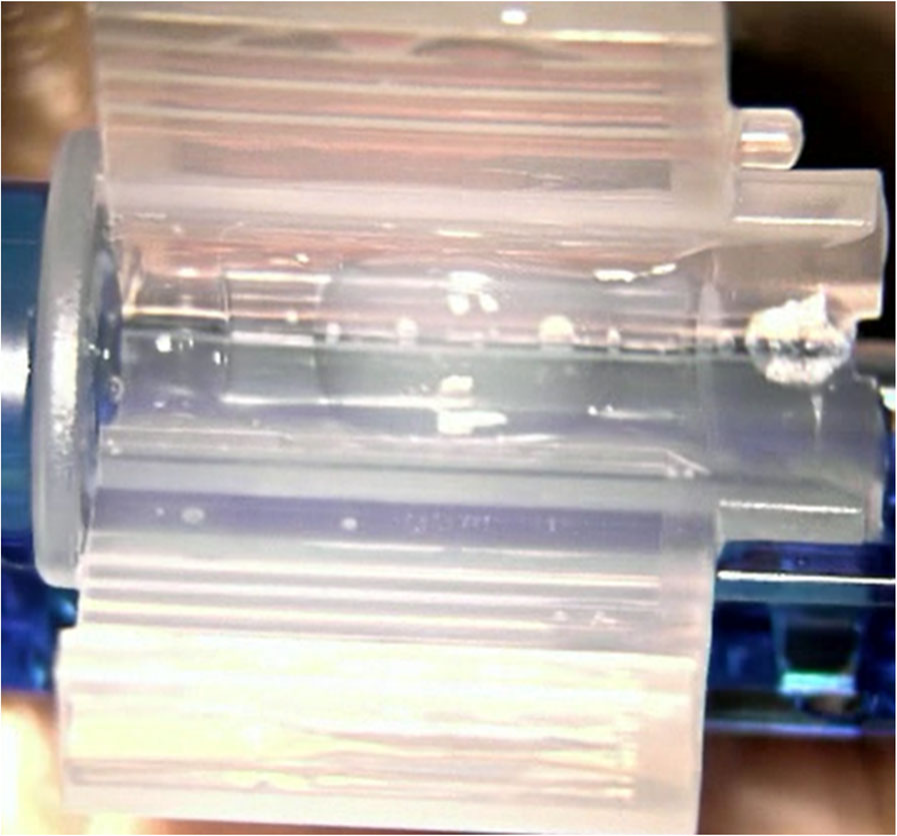
AT LISA tri 839mp is tansparent before implantation

**Fig. 2:**
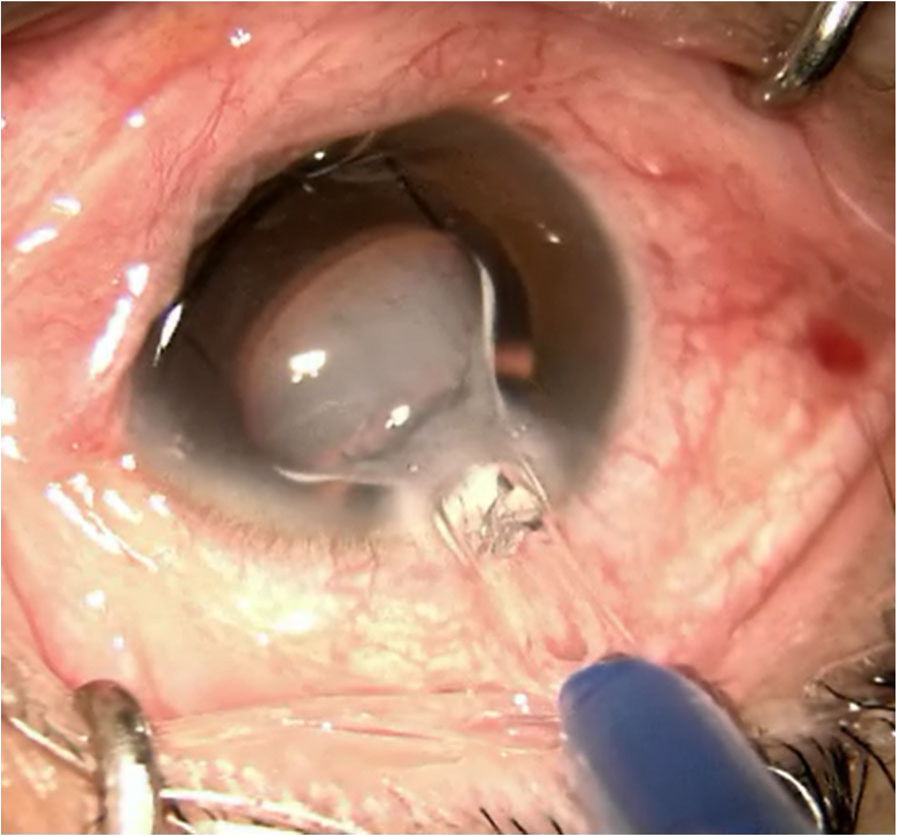
The optical region was cloulding immediately as soon as implanted into eye

**Fig. 3:**
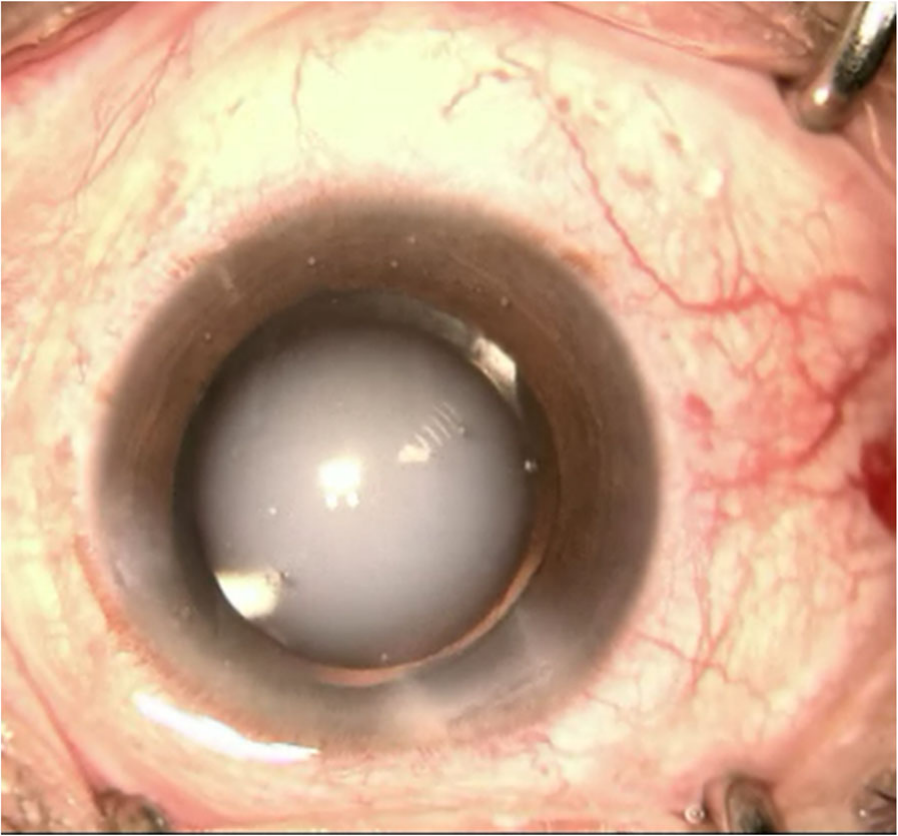
The first IOL kept cloudy persistently without alleviation for 1 h in vivo

**Fig. 4:**
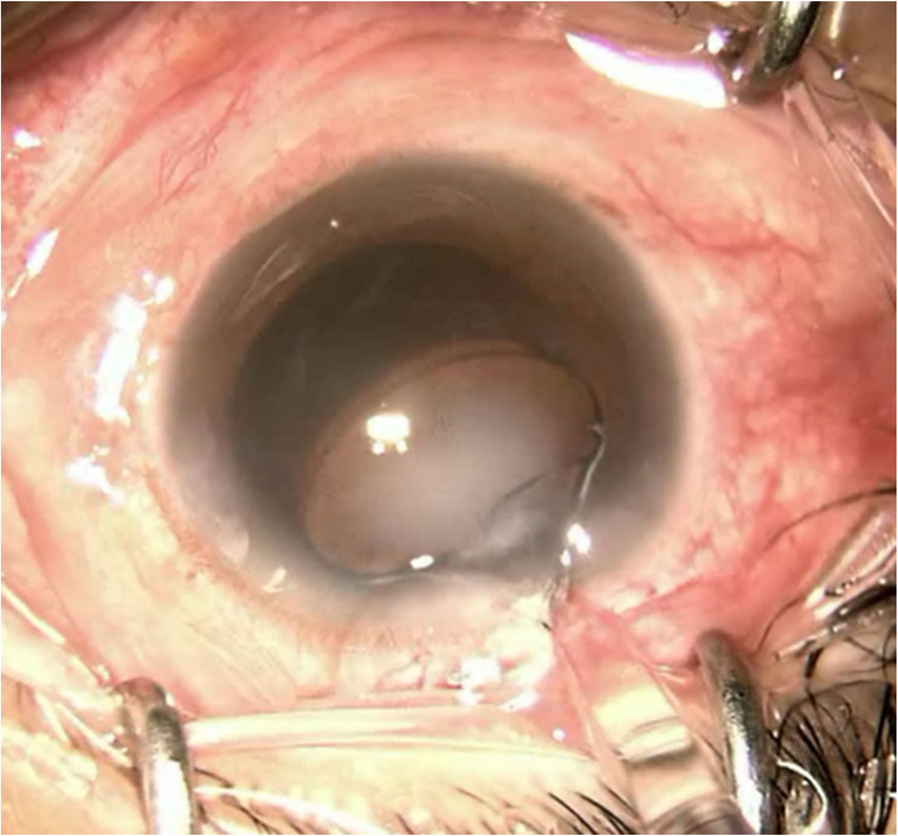
The second AT LISA tri 839mp turned cloudy immediately as implanted into eye

**Fig. 5:**
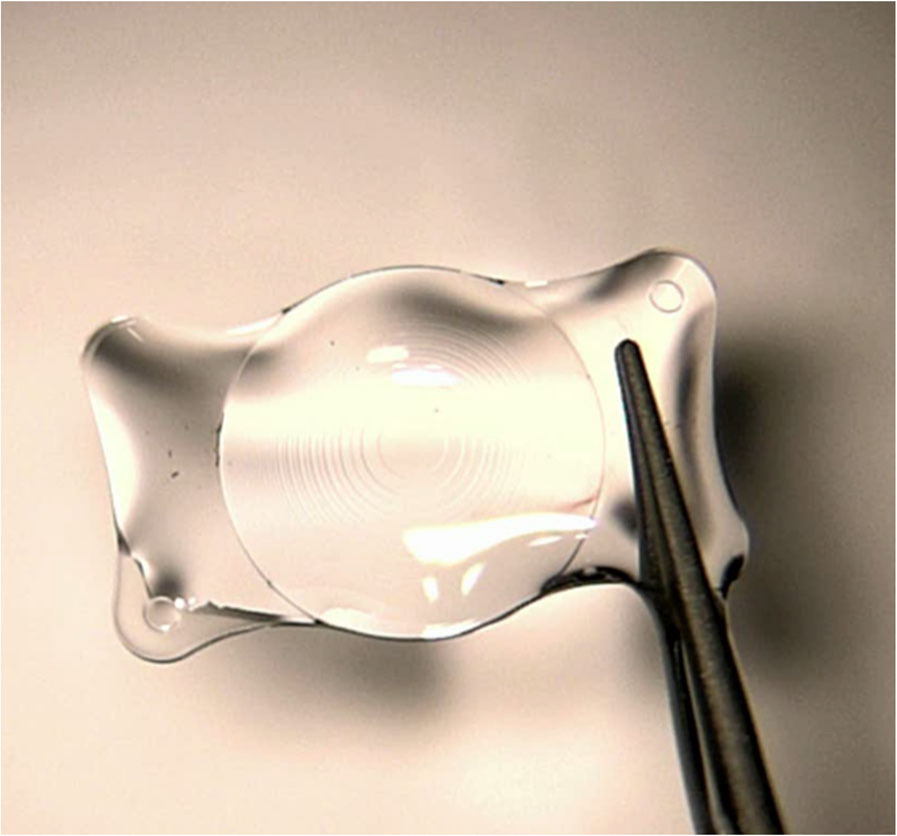
The second IOL became partially transpearent 5 min after explantation

In this patient, we routinely used perioperative regimen of mydriasis by tropicamide 4 times every 10 min, superficial anesthesia by proparacaine 4 times, Medical Sodium Hyaluronate Gel(17 mg/ml, Bausch& Lomb), and compound chloride perfusate. After lens fragmentation using femtosecond laser, phacoemulsification was uneventful and carried out under topical anesthesia. The IOL was transparent before implantation (Fig. 1). However, as soon as the optical region was implanted into the eye, it became cloudy (Fig. 2), and remained cloudy without alleviation (Fig. 3) for 1 h in vivo(Waiting for the second +18.5D AT LISA tri 839mp,we observed whether the clouding IOL would be clear). The IOL was removed using small incision on the sclera. We try another AT LISA tri 839mp lens, which was transferred from local company outside, The temperature outside was about −3 °C. The IOL had been in the theater for about 10 min before implantation into the eye. The same phenomenon occurred (Fig. 4),the IOL kept cloudy for 8 min in vivo then moved out. It became transparent 5 min later in vitro (Fig. 5). After acquiring of the patient’s informed consent, a ZCB00(Allergan) was finally implanted for safety. The post operative day 1 uncorrected visual acuity was logMAR 0.1,best corrected visual acuity was also logMAR 0.1.

## Discussion

To meet the ever increasing demand of vision quality, more and more high-end IOLs were applied in refractive cataract surgery. AT LISA tri 839mp, as a kind of trifocal IOLs, could provide distant, medium, and near vision.

Femtosecond laser technology has the ability to facilitate and optimize manual portions of cataract surgery [10–12]. Zhang S [13] reported that inflammatory cytokines IL-1β, IL-6, and PGE2 significantly increased after femtosecond laser-assisted cataract surgery. However, to the best of our knowledge, there was no report about IOL opacification caused by femtosecond laser and correlative inflammatory cytokines.

Different from the lenses (hydrophilic acrylic) that were previously reported to become cloudy in vivo, AT LISA tri 839mp IOL is made up of hydrophilic acrylic (25%) with hydrophobic surface properties. As Yu AK reported, the pseudocataracts occurred spontaneously due to calcium and phosphate accumulation, which resulted in hydroxyapatite crystal formation [14]. The surface of this trifocal IOL is made of hydrophobic acrylic, and the opacification appeared immediately after implantation, which may not cause any chemical, mechanical, or geometric change to the IOL polymer.

Similar to the report by Pallavi Tyagi [9], the IOL we used was delivered to the theater (about 20 °C) shortly before the procedure from the store located outside the premises. The outdoor temperature was about −3 °C. The IOL had been in the theater for only15 min before implantation into the eye. The opaqueness in Pallavi Tyagi’s report lasted for about 3 h then cleared, and the opacification reported by Sezer Helvacı [15] disappeared by the following day. The first AT LISA tri 839mp remained cloudy without alleviation for about 1 h,then moved out. The second one became opacified again as soon as the optical region implanted into the eye. However,5 min after the second IOL was moved out, it became clear. The two IOLs underwent the same temperature changes. Moreover, the manufacturer’s manual mentioned that opacification can occur due to a change in temperature. We deduced that the change of temperature caused the opacification of AT LISA tri 839mp in this report.

## Conclusion

The acute intraoperative clouding of trifocal lens (AT LISA tri 839mp) was caused by fluctuation of temperature in a short time. Therefore, we suggest that the IOL’s temperature should be kept in normal range as manufacture’s guideline requested (2–45 °C) before implantation. This may avoid IOL acute clouding caused by abrupt change of temperature (less than 2 °C to anterior chamber temperature) during implantation especially during winter season in the countries in temperate or polar zone.

## Abbreviations

IL-1β: Interleukin-1 beta
IL-6: Interleukin-6
IOL: Intraocular lens
IOLs: Intraocular lenses
logMAR: Logarithm of Mininal Angle Resolution
PGE2: Prostaglandin E2
